# A common signature of brain metastases among patients with breast cancer, melanoma, and lymphoma

**DOI:** 10.1093/noajnl/vdag125

**Published:** 2026-05-10

**Authors:** Thuy Thi Nguyen, Duc Manh Hoang, Tai Van Nguyen, Frédéric Pamoukdjian, Li Wang, Maxime Battistella, Catherine Miquel, Céleste Lebbé, Quang Van Le, Wei-Li Zhao, Anne Janin, Jean-Paul Feugeas†, Guilhem Bousquet†

**Affiliations:** National Cancer Hospital, Ha Noi, Vietnam; SynKoMIC Research Unit, Université Sorbonne Paris Nord, Villetaneuse, France; Hanoi Medical University, Ha Noi, Vietnam; National Cancer Hospital, Ha Noi, Vietnam; SynKoMIC Research Unit, Université Sorbonne Paris Nord, Villetaneuse, France; Hanoi Medical University, Ha Noi, Vietnam; National Cancer Hospital, Ha Noi, Vietnam; SynKoMIC Research Unit, Université Sorbonne Paris Nord, Villetaneuse, France; Hanoi Medical University, Ha Noi, Vietnam; SynKoMIC Research Unit, Université Sorbonne Paris Nord, Villetaneuse, France; APHP, Hôpital Avicenne, Médecine Gériatrique, Bobigny, France; Shanghai Institute of Hematology, State Key Laboratory of Medical Genomics, National Research Center for Translational Medicine at Shanghai, Ruijin Hospital, Shanghai Jiao Tong University School of Medicine, Shanghai, China; Pôle de Recherches Sino-Français en Science du Vivant et Génomique, Laboratory of Molecular Pathology, Shanghai, China; Université Paris Cité, INSERM, Paris, France; Pathology Department, APHP, Hôpital Saint Louis, Paris, France; Université Paris Cité, INSERM, Paris, France; Université Paris Cité, INSERM, Paris, France; APHP, Dermatolo-Oncology, Hôpital Saint Louis, Paris, France; National Cancer Hospital, Ha Noi, Vietnam; Hanoi Medical University, Ha Noi, Vietnam; Shanghai Institute of Hematology, State Key Laboratory of Medical Genomics, National Research Center for Translational Medicine at Shanghai, Ruijin Hospital, Shanghai Jiao Tong University School of Medicine, Shanghai, China; Pôle de Recherches Sino-Français en Science du Vivant et Génomique, Laboratory of Molecular Pathology, Shanghai, China; Université Paris Cité, INSERM, Paris, France; Université Marie et Louis Pasteur, CHU Besançon, Inserm UMR 1098, CIC 1431, Besançon, France; SynKoMIC Research Unit, Université Sorbonne Paris Nord, Villetaneuse, France; Assistance Publique Hôpitaux de Paris, Hôpital Avicenne, Service d‘Oncologie Médicale, Bobigny, France

**Keywords:** brain metastases, genomic signature, KLK6, seed and soil theory

## Abstract

**Background:**

Despite considerable improvement in cancer treatments, brain metastases are still challenging daily practice in oncology. Their biology remains largely unknown with limited data obtained from brain metastatic samples. Here, using metastatic samples of three different cancer types—breast cancer, melanoma and lymphoma—we aimed to identify a common genomic signature related to metastatic localizations in the brain.

**Methods:**

Using samples from 133 patients with metastatic breast cancer, melanoma, or lymphoma, laser-microdissection of cancer cells and transciptomic analyzes were performed on 29 brain metastatic samples, and compared to data from 104 extra-cerebral metastases. To obtain murine models of the common brain metastatic signature observed in patients with these three cancer types, *in vivo* experiments were performed after intracardiac injections of cancer cell lines.

**Results:**

Among patients, we identified 23 common genes up-regulated in brain metastases from breast cancer, melanoma, and lymphoma, including KLK6, a serine protease with trypsin-like properties and physiological expression in oligodendrocytes and normal brain endothelial cells. At protein level, KLK6 expression was significantly higher in brain metastases than in extra-cerebral metastases. In particular, KLK6 was not overexpressed in extra-cerebral metastases of patients who developed brain metastases. In murine models of breast cancer, melanoma, and lymphoma brain metastases, we confirmed that KLK6 overexpression was linked to the implantation of cancer cells in the brain.

**Conclusion:**

KLK6 overexpression is linked to brain localizations whatever the cancer type, which provides new perspectives for the development of anti-KLK6 therapeutic strategies, pending specific cancer cell targeting to avoid cytotoxicity on normal brain cells.

Key Points

- This is a transcriptomic analysis on laser-microdissected metastatic samples from three different cancer types: breast cancer, melanoma, and lymphoma.
- KLK6 overexpression is linked to brain localization, regardless of the organ of origin.

Importance of the Study

This is a transcriptomic analysis on a large series of 133 laser-microdissected metastatic samples from three different cancer types: breast cancer, melanoma, and lymphoma. Our approach is original since most studies have focused on a single cancer type. Here, we identified a 23-gene common brain signature of overexpressed genes, which could relate to an adaptive process of metastatic cancer cells, mimicking the “normal biology” of the metastasized organ, facilitating their implantation. In patients and in murine models, we demonstrated that KLK6 overexpression was linked to brain metastatic implantation in the three cancer types. In preclinical models, a higher *KLK6* expression in brain metastases was also associated with a larger brain metastatic surface area, suggesting that KLK6 overexpression not only contributes to the adaptive brain implantation of cancer cells, but also to the progression of the disease itself.

Brain metastases occur in 10% to 20% of patients with metastatic cancer, mainly deriving from lung cancer (20%-36%), primary breast cancers (15%-25%), or melanomas (5%-20%).^1–4^ In the last 20 years, the incidence of brain metastases has increased, as a result of improved control of the localizations outside the central nervous system, and because most anti-cancer drugs do not readily cross the blood-brain barrier.^5^ Despite therapeutic advances, the prognosis for brain metastasis has remained poor, with a median survival of fewer than 16 months,^6^ thus challenging practice in oncology. Considering this mainly as a compartmental limitation, several approaches have been developed to facilitate the crossing of the blood-brain barrier (bi-specific antibodies using physiological transporters, intranasal route of drug administration)^5^ or to overcome this difficulty through direct intrathecal drug administration.^7^^,^^8^

Knowledge of the biology of brain metastasis is required to optimize treatment. However, this area of biology has remained largely unexplored with limited data obtained from brain metastatic samples, mainly because of the difficult access to these localizations.^9^ Although primary tumors and metastases can harbor a common genomic signature, significant discrepancies can be identified between matched samples from a given patient, as metastatic cells can derive from a minority clone within the primary tumor.^10^^,^^11^ This may also be true for extra-cerebral metastases and brain metastases. Regarding mutational profiles, we recently performed a meta-analysis on genomic data from 1485 samples of breast cancer brain metastases and compared them to 17 447 extra-cerebral metastases and primary tumors, with significant differences in gene mutation prevalence.^12^ Transcriptomic data is even more sparse, usually obtained from extra-cerebral samples, and thus restricted to signatures predicting the risk of brain metastasis occurrence.^13–15^ In 2 recent studies performed on laser-microdissected cancer cells from lymph-node metastases of patients with metastatic breast cancer or melanoma, using transcriptomic analysis, we identified biomarkers associated with the risk of brain metastases.^13^^,^^14^

For the purpose of this research program, we thus proposed an original approach. From metastatic samples of 3 different cancer types—breast cancer, melanoma and lymphoma—we aimed to identify a common genomic signature related to metastatic localizations in the brain.

## Methods

### Metastatic Samples from Patients

One hundred and thirty-three patients with available metastatic biopsy samples from either brain metastases or extra-cerebral metastases were included in the study. Patients with breast cancer and melanoma were followed at Saint-Louis Hospital (Paris, France), and patients with non-Hodgkin lymphoma were followed at Rui Jin Hospital (Shanghai, China). All patients had metastatic breast cancer, metastatic melanoma, or stage-IV non-Hodgkin lymphoma according to Ann Arbor staging.^16^ For breast cancer and melanoma extra-cerebral metastases, data had previously been reported, enabling biomarkers associated with brain metastatic risks to be identified.^13^^,^^14^

At the time of biopsy sampling, all patients had undergone whole-body computed tomography within the last month, including brain imaging.

In compliance with French bioethics legislation (2004-800; June 8, 2004), all patients had been informed of the research use of the part of their samples remaining after diagnosis had been established, and none opposed it. Informed consent was obtained from each patient. The Clinical Research Board Ethics Committee approved this study (CPP-Ile-de-France#13218).

On the basis of clinical and imaging data, we separated patients with and without brain metastases. Brain metastasis samples were stored in the Sainte-Anne Hospital tumor bank and other metastatic samples were deposited in the Saint-Louis Hospital tumor bank. All dissections and RNA extractions were conducted at a single site in French research unit, according to the protocol described below.

### Laser-Microdissection of Cancer Cells and RNA Extraction for Transcriptomic Analyzes

All metastatic samples were laser-microdissected to exclusively select cancer cells and prevent contamination between the tumor microenvironment and normal tissue.

All 133 patients had at least one frozen metastatic biopsy available. For each sample, 7 µm cryo-cut sections were laser-microdissected to select a minimum of 1500 tumor cells for a minimum surface area of 471 000 µm^2^, using a PALM Microbeam/Zeiss-system. Total RNA was extracted from the laser-microdissected tumor cells using RNeasy-Mini-Kit (Qiagen, France), quantified on NanoDrop and qualified on Bio-RadExperionTM Automated-Electrophoresis-Station (BioRad, France).

Transcriptomic analyzes were performed using MiltenyiBiotec-Microarray service. A linear T7- based amplification step was performed on 0.5 μg of all RNA samples. To produce Cy3-labeled cRNA, the RNA samples were amplified and labeled using an Agilent-Quick-labelling kit. Yields of cRNA and dye-incorporation rates were measured with a ND-1000-Spectrophotometer (NanoDrop, LabTech, France). Hybridization was performed according to the Agilent 60-mer-oligomicroarray protocol: 1.65 μg Cy3-labeled cRNA were hybridized (overnight/65 °C) on Agilent-Whole-Human-Genome-Oligo-Microarrays 8 × 60K V2, and fluorescence signals detected using AgilentMicroarray-Scanner. Agilent-FE-Software determined the feature intensities. Quantile normalization was performed using the limma package on R-software version 4.1.0 (Foundation for Statistical Computing, Vienna, Austria), based on log2 single-intensity expression data. To account for technical effects related to array design and RNA quality, the merged gene-level matrix was adjusted using a gene-wise linear model implemented in limma, via the remove Batch Effect function in R. The effect of the RNA integrity number (RIN) was removed as a continuous covariate, although RIN did not significantly vary by cancer type (Supplementary Figure S1).

### Genes Selected for Analyzes

#### RT-qPCR and validation of mRNA expression in metastatic samples

On following sections of the same laser-microdissected metastatic samples, we used RT-qPCR (reverse transcription quantitative real-time PCR) to validate the transcriptomic results. Total RNA was reverse-transcribed (cDNA) before qPCR amplification using random primers with SuperScriptTM-II-Reverse Transcriptase (Invitrogen, France). The qPCR reactions were performed using fluorescent probes on a CFX96 Real-Time-System (Bio-Rad) according to the MIQE guidelines.^17^ A blank sample with no cDNA was included, and the experiments were performed in triplicates for *KLK6* [Hs00160519_m1], *AQP4* [Hs00242342_m1] genes, with each sample duplicated on the PCR plate. The 2 reference genes *TBP* [Hs00427620_m1] and *GAPDH* [Hs02786624_g1] were used to normalize gene expression results. The results were expressed as dCt and RQ (relative quantification) using the 2^−ΔΔCt^ method.^18^

#### KLK6 expression in metastatic samples

Using immunohistochemistry, KLK6 expression was assessed in the brain metastatic samples and the extra-cerebral metastatic samples of the 133 patients. An indirect immunoperoxidase method (Discovery/RocheDiagnostics) on 5 µm-thick frozen tissue sections was performed using anti-KLK6 (clone E24, monoclonal antibody, Merck Millipore) as a primary antibody. The secondary anti-mouse antibody was provided by the DABMAP detection kit (Roche-Diagnostics). Systematic controls were the absence of a primary antibody and the use of an irrelevant primary antibody of the same isotype.

For each tissue section, cells expressing KLK6 were counted by 2 different pathologists (G.B. and A.J.) on 5 different fields at ×400 magnification, using a ProvisAX70 microscope (Olympus, Tokyo) with a wide-field eyepiece number 26.5, providing a field size of 0.344 mm^2^ at this magnification.

Membranous and cytoplasmic distribution of KLK6 was considered positive. For each field, a minimum of 100 tumor cells was analyzed. The percentage of KLK6-expressing cells was the number of positive cells in these 100 tumor cells. Each sample was given a score by multiplying the stain intensity grade (0 = no staining, 1 = low intensity, 2 = medium intensity, 3 = strong intensity) by the numerical code for the percentage of positive cells (0 = 0%, 1 = under 10%, 2 = 10%-50%, 3 = 51%-80%, 4 = over 81%). The maximum score was 12 when more than 81% of the cells expressed KLK6 with a strong intensity signal. Results were expressed as means ± standard errors (SE).

### Breast Cancer, Melanoma, and Lymphoma Cell Lines

We chose 3 murine cell lines and one human cell line for their brain metastatic potential following intracardiac injection.^19–25^ The 4 cell lines were obtained from ATCC (Rockville, MD, United States).

The 3 murine cell lines were the triple-negative breast cancer cell line 4T1, the melanoma cell line B16-F10 and the lymphoma T cell line S49.1. The human cell line was MDA-MB-231, which is a triple-negative breast cancer cell line. 4T1 cells are derived from a Balb/c breast tumor, B16-F10 are derived from C57BL/6J cutaneous melanoma, and S49.1 are derived from the thymus of a Balb/c. We purposely chose triple negative breast cancer cell lines (human cell line MDA-MB-231 and murine cell line 4T1) since in patients brain metastases almost only occur with triple negative or HER2-overexpressed subtypes. In addition, triple-negative breast cancers are not estrogen dependent, justifying the fact that we did not use estrogen supplementation.

Cell lines were cultured in a humidified atmosphere containing 5% CO_2_ at 37 °C. 4T1 cells were grown in RPMI high glucose (Gibco), 10% fetal bovine serum (PAA), L-glutamine 4 mM (Sigma Aldrich), 1% penicillin/streptomycin (Sigma Aldrich). B16-F10 cells were grown in DMEM high glucose (Gibco), 10% fetal bovine serum (PAA), L-glutamine 4 mM (Sigma Aldrich), 1% penicillin/streptomycin (Sigma Aldrich). S49.1 cells were grown in DMEM high glucose (Gibco), 10% horse serum (PAA), 1% penicillin/streptomycin (Sigma Aldrich). MDA-MB-231 cells were grown in DMEM high glucose (Gibco), 20% horse serum (PAA), 1% penicillin/streptomycin (Sigma Aldrich).

### Murine Models of Brain Metastases

For *in vivo* experiments, six-week-old NMRI nude mice, maintained in specific pathogen-free conditions, received an intracardiac injection with 50 000 cells to obtain brain metastatic localizations (*N* = 5 animals for each cell line).

The University Institute Board Ethics Committee for experimental animal studies approved this study (APAFIS #47139-202312151436769), and the animal housing of the research facility was certified (Agreement D93-008-01). The mice were monitored, with daily observations of weight loss, grooming behavior, posture, respiratory rate, and activity. When signs of suffering were observed, the mice were euthanized by cervical dislocation.

At the time of euthanasia, for each mouse, all organs were collected to assess the metastatic spread. In particular, the whole brain was removed and cut longitudinally into 2 parts, one immediately snap-frozen and 1 formalin-fixed.

Assessment of the brain metastatic spread was performed on 2 µm slides stained with haematoxylin-eosin. Each slide was scanned using a Nanozoomer 2.0 HT scanner (Hamamatsu, Japan). For each mouse, brain metastatic areas were delineated on 4 consecutive virtual slides and quantified using DotSlide software. At the time of euthanasia, for each mouse, the mean surface area of the brain metastatic extension was calculated as the percentage (%) of the surface of brain metastases and the surface of the whole brain.

### Assessment of KLK6 Expression on Mouse Brain Metastases and Extra-Cerebral Metastases

For each sample (brain metastases and extra-cerebral metastases), 7 µm-thick tissue sections were laser-microdissected to select a minimum of 1500 tumor cells for a minimum surface area of 430 000 µm^2^, using a PALM-Microbeam/Zeiss-system (Carl Zeiss, Germany).

*KLK6* mRNA expression was then assessed using RT-qPCR as described previously.

### Murine Oligodendrocytes Obtained from the Brains of NMRI Nude Mice

Immediately after euthanasia of a nude mouse by cervical dislocation, the brain was removed and placed in PBS. After mechanical dissociation using GentleMACS, the brain, previously cut into 2 mm fragments, was dissociated enzymatically using a Tumor Dissociation Kit (Miltenyi, Biotec). A first wash was performed in PBS MgCa-free with 0.9 M sucrose after successive filtration on 70 µm then 40 µm. The supernatant containing the myelin was then removed, and a second wash was performed in PBS MgCa-free with 4% BSA. After removal of the supernatant, the pellet is resuspended in DMEM/F12 medium supplemented with horse serum 20%, insuline 5 µg/mL, hydrocortisone 20 µg/mL, sodium pyruvate 110 mg/mL, and B27 neuromix 1X). After 24 hours of culture, the astrocytes adhered to the support, and the oligodendrocytes that did not adhered were harvested and cultured in the same medium using poly-L-lysine-coated wells (0.1 mg/mL, BioReagent, Merck).

### Oligodendrocyte Characterization Using Immunostaining and RT-qPCR

For immunofluorescence staining, oligodendrocytes were grown separately on culture slides (#354104; BD Falcon, BD Biosciences). Then, an indirect immunofluorescence method was run using rabbit anti-murine Olig1 monoclonal antibody (AB-24.0011, Clone E3T6D, 1/100 OrbmCT32, US) or rabbit anti-murine Olig2 monoclonal antibody (AB-24.0012, Clone E6G6Q, 1/100 OrbmCT32, US) as primary antibodies, Olig1 and Olig2 being specific markers of oligodendrocyte cell maturation. A chicken anti-rabbit FITC antibody (−ab6825-1) was used as secondary antibody, and fluorescent mounting medium with DAPI was used for nucleus detection (ABCAM—ab104139). The fluorescence staining was observed at 400× magnification on a BX63 microscope (Olympus).

Using RT-qPCR, we assessed the mRNA expression level of *Olig1 Mm* and *Olig2* from oligodendrocytes cultured alone or cocultured with 4T1 cells. 4T1 and S49.1 cells were used as negative controls.

### 4T1 Cocultured with Murine Oligodendrocytes or Using Conditioned Medium

For coculture assay, we used Boyden chambers on 6-well plates with membrane pores of 1 μm size (Millipore PIRP30R48), and murine oligodendrocytes were first seeded on the lower part. At confluence, 10^5^ 4T1 cells were seeded in the upper chamber. After 3 days, the 2 different cell types were collected for *KLK6* mRNA expression assessment. Four other conditions were tested: (1) 4T1 cells cultured alone in DMEM/F12 medium; (2) 4T1 cells cultured alone in oligodendrocyte medium; (3) 4T1 cells cultured alone with oligodendrocytes supernatant; (4) oligodendrocytes alone cultured in the specific medium.

### Statistical Analyzes

All analyzes were conducted in R (v4.1.0; R Foundation for Statistical Computing, Vienna, Austria; https://www.R-project.org).

Quantitative variables were expressed as means ± standard deviations (SD) and categorical variables as numbers and percentages.

#### Unsupervised analysis

To summarize the gene expression dataset, we first performed a principal component analysis (PCA). PCA identifies principal components, which are linear combinations of the original variables.^26^ Its objective is to reduce dimensionality by defining a limited number of axes that explain the largest possible proportion of variance in the gene expression dataset. In our analysis, the largest proportion of variance among metastasis samples was attributable to the primary tumor of origin (Supplementary Figure S2).

#### Transcriptomic data gene selection workflow

To limit confounding and identify genes preferentially upregulated in brain metastases, we used a two-step selection strategy (Supplementary Figure S3).

- Within the brain-metastasis subset, we first removed genes whose expression differed across the 3 cancer types (breast cancer, melanoma, and lymphoma) using a Kruskal-Wallis test with Bonferroni correction.
- We then assessed differential expression between brain and extra-cerebral metastases separately within each cancer type using significance analysis of microarrays (SAM), which relies on a modified t statistic and permutation-based control of the false discovery rate (FDR) usually called q-value (R, SAMR package). The q-value was computed empirically by permuting sample labels to generate a null distribution of test statistics and comparing it with the observed data. We retained only differentially expressed genes with significant q values (based on 1000 permutations performed for each tumor type).

To complete this workflow, we fitted 2 multivariable regression models to all genes in the transcriptomic dataset (1) a logistic regression model with metastasis location (brain vs extra-cerebral) as the outcome and gene expression as the main predictor, adjusted for tumor type and center (binomial glm in R); and (2) a Gaussian linear regression model with gene expression as the outcome and metastasis location (brain vs extracerebral) as the main predictor, adjusted for tumor type and center (Gaussian glm in R). *P* value were corrected across genes using the false discovery rate (FDR) method (p.adjust function with method = “fdr” in R). These multivariable analyzes consistently confirmed the findings of our initial study and allowed us to retain all genes selected in the initial two-step analysis (Supplementary Table S1A and B and Supplementary Figure S4).

#### External datasets

We performed similar multivariate analyzes (Gaussian and binomial models) using publicly available data downloaded from the NCBI GEO database. We were able to use the following studies, in which the authors collected samples of brain or extra-cerebral metastases derived from breast cancer: GSE14020 (microarray study comparing gene expression profiles of 29 breast cancer metastases across different organs), GSE209998 (RNA-seq data from 44 primary breast tumors and their matched metastases, including 9 brain metastases and 67 metastases at other sites), GSE1913103 (RNA-seq data from 12 brain metastases and 65 metastases at other sites), GSE184717 (RNAseq study focusing on a single patient with primary breast cancer and multiple metastases). Across the 4 datasets, per-gene fixed-effects meta-analysis was performed using inverse-variance weighting. For each gene g and each dataset i, we extracted the estimated log_2_ fold change (FC_g, i_) and its standard error (SE_g, i_) from the multivariate Gaussian linear model. Weights were defined as wg,i = 1 / SE2g,i. The pooled fixed-effect (FE) estimate was computed as $\log_{2}{FC}_{g,FE}=\sum_{i}w_{g,i}\log_{2}{FC}_{g,i}/\sum_{i}w_{g,i}$, with pooled standard error ${SE}_{g,FE}={\left(\sum_{i}w_{g,i}\right)}^{-1/2}$. Two-sided Wald Z-tests (Z = log2 FCg,FE / SEg,FE) were used to provide pooled *P*value and 95% confidence intervals ($\log_{2}{FC}_{g,FE}\pm {SE}_{g,FE}$).

#### Other statistical analyzes

The Mann-Whitney test was used to compare *KLK6* or *AQP4* mRNA expression levels in the breast cancer, melanoma and non-Hodgkin lymphoma metastatic samples.

Correlations between KLK6 mRNA expression and the mean surface area of brain metastases for the corresponding mouse model were assessed using Pearson’s correlation test (R) quantitative variables were expressed as means ± standard deviations (SD) and categorical variables as numbers and percentages.

#### Data availability and R code

The raw and processed data, as well as the R code used in this study, are available at the following Zenodo repository: https://doi.org/10.5281/zenodo.18678201.

## Results

### Characteristics of the 133 Patients With Transcriptomic Analyzes of Metastatic Samples

**Figure 1.**
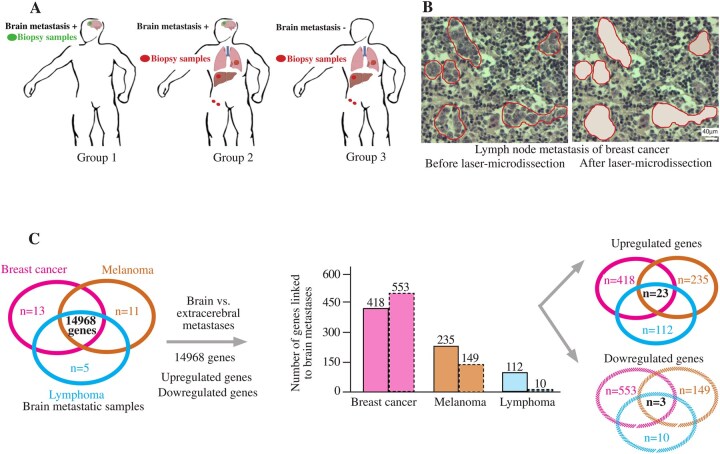
Analytical approach for transcriptomic data from metastasis biopsies. (A) Group 1 includes patients with brain metastases and a brain metastatic sample; group 2 includes patients with brain metastases and an extra-cerebral metastatic sample; group 3 includes patients without brain metastases, and an extracerebral metastatic sample. (B) In a breast cancer brain metastasis, laser microdissection enables the specific selection of cancer cells surrounded by inflammatory cells. (C) Across brain metastases from breast cancer, melanoma, and lymphoma, 14 968 common genes were not significantly differentially expressed among the 3 cancer types. Among these 14 968 common genes, comparisons between brain metastasis samples and extracerebral metastasis samples for each cancer type identified 23 common genes upregulated in brain metastases from breast cancer, melanoma, and lymphoma (and 3 downregulated genes).

Among the 133 patients with metastatic cancer, 58 had breast cancer, 62 had melanoma, and 13 had non-Hodgkin lymphoma. Patients were divided into 3 groups according to the type of metastatic sample analyzed, and according to the presence or absence of brain metastases: group 1 included patients with brain metastases and brain metastatic samples; group 2 included patients with brain metastases and extra-cerebral metastatic samples; group 3 included patients without brain metastases, and with extra-cerebral metastatic samples (Figure 1A). Most extra-cerebral metastatic samples were lymph node metastases (*N* = 88), or liver metastases (*N* = 14) (Table 1). Each of the 133 metastatic samples was laser-microdissected to select at least 1500 cancer cells for further transcriptomic analyzes (Figure 1B). After RNA extraction, all samples were good quality, with a mean RNA integrity value of 8.8 (range: 6.1-10) (Supplementary Figure S1).

**Table 1.** Distribution of the 133 patients with transcriptomic analyzes of metastatic samples

		Breast cancer *N* = 58	Melanoma *N* = 62	Non-Hodgkin lymphoma *N* = 13
Group 1	*Patients with brain metastasis* *Biopsy samples of brain metastasis*	*N* = 13	*N* = 11	*N* = 5
Group 2	*Patients with brain metastasis* *Biopsy samples of extra-cerebral metastasis*	*N* = 18 Lymph nodes = 13 Lung = 3 Liver = 4	*N* = 18 Lymph nodes = 18	*N* = 0
Group 3	*Patients without brain metastasis* *Biopsy samples of extra-cerebral metastasis*	*N* = 27 Lymph nodes = 16 Liver = 10 Lung = 1	*N* = 33 Lymph nodes = 33	*N* = 8 Lymph nodes = 8

### A Common Signature for Brain Metastases from Patients With Breast Cancer, Melanoma or DLBCL

Using transcriptomic data obtained from 133 metastatic samples, we first performed a factor analysis of mixed data that incorporated qualitative variables (tumor type and patient group) and quantitative transcriptomic variables. We conducted an unsupervised PCA. The main sources of variation (Dimensions 1 and 2 of the PCA) clustered metastases according to the tumor of origin (breast, lymph nodes, or skin), independently of metastatic site (Supplementary Figure S2).

We analyzed brain metastatic samples from the breast cancers (*N* = 13), melanomas (*N* = 11), and non-Hodgkin lymphomas (*N* = 5), in order to remove genes not significantly differently expressed across these 3 cancer types. Using a Kruskal-Wallis test and a significance threshold for a *P* value of 5%, we retained 14 968 common genes (Figure 1C, Supplementary Figure S3A). We then performed SAM within each tumor type to identify genes upregulated in brain-metastasis samples, regardless of their origin. We observed this pattern when analyzing 14 968 genes within each cancer type, comparing brain-metastatic with extra-cerebral metastatic samples. We retrieved 23 common genes up-regulated in the brain metastases of breast cancers, melanomas, and lymphomas (Figure 1C, Supplementary Figure S3B, and Table 2). We also identified 3 downregulated genes (*H19, CCL19, and MEOX1*); however, we focused exclusively on upregulated genes in order to highlight biomarkers positively associated with brain metastases.

**Table 2.** Common genes up-regulated in brain metastases of breast cancer, melanoma, and non-Hodgkin lymphoma

			Breast cancer		Melanoma		Lymphoma	
Gene	Rank	Median SAM score	Log_2_ FC	SAM score	Log_2_ FC	SAM score	Log_2_ FC	SAM score
*NKX6-2*	1	8.6	5.2	8.6	5.6	10.9	6.5	5.0
*GFAP*	2	8.4	3.7	10.4	2.9	8.4	4.3	3.9
*STMN4*	3	6.9	3.3	6.9	3.7	9.0	4.9	4.8
*SOX2-OT*	4	5.9	2.4	5.8	3.1	6.3	5.0	5.9
*LOC284570*	5	5.7	2.1	6.0	2.4	5.7	3.4	4.2
*GRM3*	6	5.5	2.6	9.2	1.8	5.5	3.4	4.5
*CNTN2*	7	5.4	3.3	5.4	5.1	8.7	5.6	3.8
*CACNG7*	8	5.3	1.4	6.3	1.2	5.3	2.0	4.1
*MT3*	9	5.3	2.3	6.4	1.6	5.3	2.4	4.9
*GPR37L1*	10	5.3	2.5	5.3	3.8	6.5	4.7	4.4
*NCAM1*	11	5.3	2.7	7.6	2.7	4.0	4.9	5.3
*FAM123A*	12	5.2	2.5	5.2	3.3	7.0	4.8	5.2
*AQP4*	13	5.2	2.7	5.2	3.1	8.7	3.0	4.2
*MOBP*	14	5.1	2.0	5.1	2.7	7.1	3.5	3.7
*CLDN11*	15	4.9	1.7	3.3	3.2	4.9	4.5	5.5
*GAP43*	16	4.5	3.3	10.2	3.1	4.5	4.7	4.5
*NCAN*	17	4.4	1.4	2.9	1.3	4.8	2.6	4.4
*DNER*	18	4.4	2.2	4.0	2.6	4.4	4.0	5.3
*TMEM229A*	18	4.3	2.0	5.2	2.5	4.3	3.2	3.9
*KLK6*	20	4.2	3.7	4.2	3.8	6.3	3,9	3,8
*OMG*	21	4,2	1,7	4,2	2,1	3,8	3,3	4,6
*KIF5C*	22	4,1	1,4	2,9	2,6	4,1	3,5	5,9
*MAG*	23	3,8	1,6	3,7	1,6	3,8	2,7	4,0

Genes passing the q-value threshold could be ranked based on their SAM d-scores, reflecting both effect size and expression stability. Log_2_ FC: Log_2_ Fold Change between brain metastases and extra-cerebral metastases.

In addition, we fitted gene-wise multivariable regression models (binomial and Gaussian linear), using either metastasis location (cerebral vs extra-cerebral) as the outcome and gene expression as the predictor, or gene expression as the outcome and metastasis location as the predictor, adjusting for tumor type and center. The models were adjusted for the center at which the patient was initially treated, included as a covariate. This analysis placed the entire 23-gene panel among the most significantly upregulated in brain metastases (Supplementary Table S2A and B, and Supplementary Figure S4). Because the generalized linear model (GLM) relies on two-sided tests without assuming a predefined direction of effect, it also identified genes downregulated in brain metastases, including the 3 downregulated genes detected by the two-step workflow. As stated above, we did not further investigate these findings, as the present study focuses on the robust characterization of upregulated markers.

As expected, when we performed a supervised PCA using exclusively the 23 genes upregulated in brain metastases, we observed a clear separation between cerebral and extra-cerebral metastases (Supplementary Figure S5).

We also examined the expression of the 23 genes in 4 published gene expression datasets of breast cancer brain metastases (GSE14020, GSE209998, GSE193103, and GSE184717) (Supplementary Table S2A-E). Twenty of these 23 genes were present in the 4 datasets (*MOBP*, *NCAN*, *KIF5C*, *CNTN2*, *SOX2-OT*, *NKX6-2*, *MT3*, *OMG*, *CACNG7*, *GFAP*, *GPR37L1*, *MAG*, *STMN4*, *CLDN11*, *AQP4*, *GAP43*, *GRM3*, *NCAM1*, *KLK6*, and *DNER*) and were confirmed to be more highly expressed in cerebral metastases than in extra-cerebral metastases.

Most of the 23 genes are involved in the development of the central nervous system. We carried out a systematic literature search for these 23 genes, to understand their potential link with brain metastases, primary brain tumors and carcinogenesis hallmarks (Supplementary Table S3). Only one gene, *GAP43,* had been reported previously to be overexpressed in lung cancer brain metastases compared to primary tumors.^27^ At least 12 of them had been described in the pathogenesis of primary brain tumors, mainly glioblastomas. Their overexpression was associated with shorter survival.^28–31^ We decided to focus on *KLK6* since there were 14 references related to carcinogenesis hallmarks in several different cancer types (Supplementary Table S3), including proliferation, migration, and invasion.^32–34^

### KLK6 Overexpression Is Linked to Metastatic Localization in the Brain

**Figure 2.**
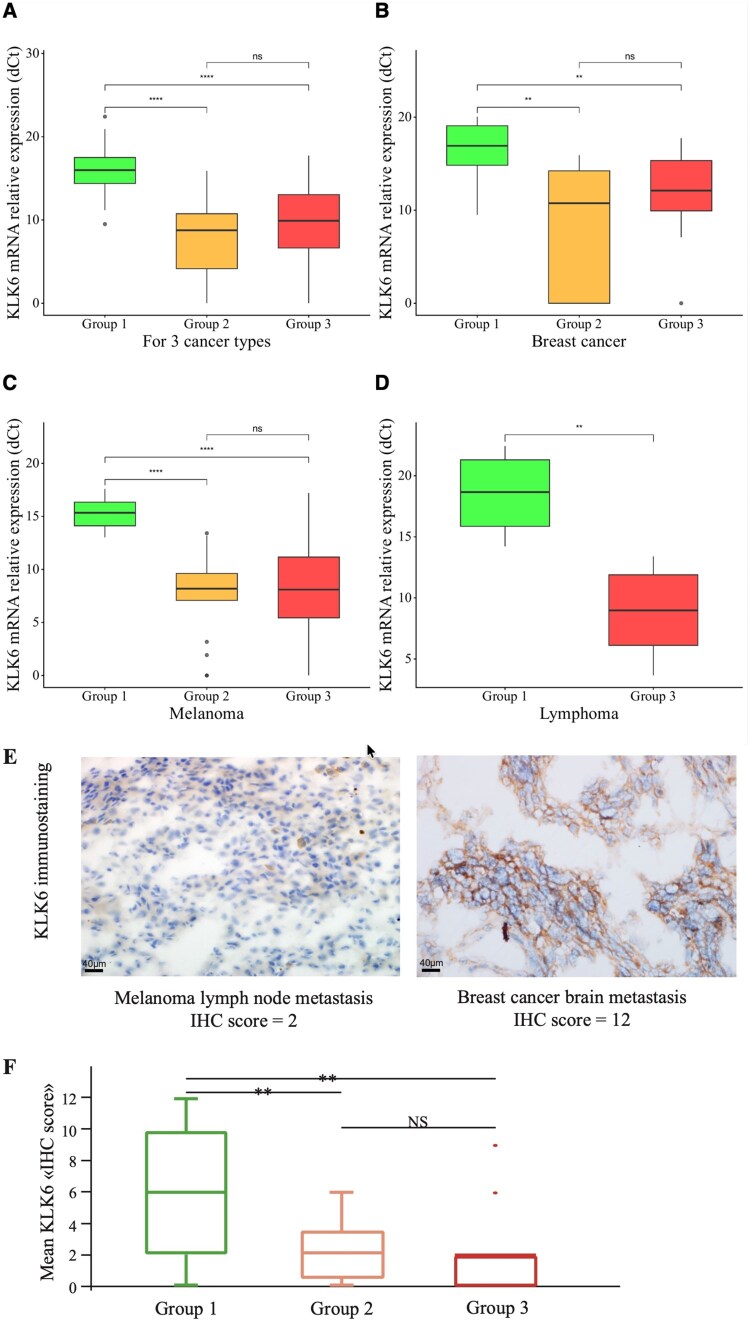
KLK6 mRNA expression according to subgroups in the 3 cancer types and KLK6 protein expression using an immunohistochemistry score. KLK6 mRNA expression among the 3 groups (A), and then separately in each cancer type (B, C, D); **P < .01, ***P < .001, ****P < .0001. Panel E shows KLK6 expression in a melanoma lymph node metastasis with a low “IHC score” of 2 (left panel), and in a breast cancer brain metastasis with a high “IHC score” of 12 (right panel). KLK6 mean “IHC score” is significantly higher in Group 1 compared to Group 2 (6.0 vs 2.1, *P* < .001), and Group 1 compared to Group 3 (6.0 vs 1.9, *P* < .001) (Panel F); **P < .001.

Using RT-qPCR, we then assessed *KLK6* expression in laser-microdissected cancer cells from all 133 metastatic samples of our initial series. We compared *KLK6* expression across the 3 groups according to metastatic localization and to the type of metastatic sample analyzed. When all 133 samples were pooled, we showed significant *KLK6* overexpression in Group 1 *versus* Group 2 or Group 3 (*P *< .001 and *P *< .05, respectively) (Figure 2A). When we analyzed each cancer type separately, we confirmed that *KLK6* expression was significantly higher in brain metastases (Group 1) than in extra-cerebral metastases (Groups 2 and 3) (Figure 2B-D). In particular, it is worth noting that KLK6 was not overexpressed in extra-cerebral metastases from patients who developed brain metastases.

To confirm the results of our transcriptomic data on individual samples, we assessed the mRNA expression of another gene significantly overexpressed in brain metastases in all 3 cancer types, *AQP4* (Supplementary Table S2A). Again, we found significant *AQP4* overexpression in Group 1 vs Group 2 or Group 3 (Supplementary Figure S6).

At protein level, we also assessed KLK6 expression from the 133 samples of brain and extra-cerebral metastases using immunohistochemistry (Figure 2E). In our study, histological analyzes were always performed by 2 pathologists (A.J. and G.B.) blinded for the cases analyzed. The inter-rater agreement was also calculated by Cohen’s Kappa (κ). In our study, Cohen’s Kappa coefficient of 0.82 indicates strong agreement between 2 pathologists. When we compared KLK6 mean scores in the 3 different groups, we showed that the KLK6 mean “IHC score” was significantly higher in Group 1 than in Group 2 (6.0 vs 2.1, *P *< .001), and in Group 1 compared to Group 3 (6.0 vs 1.9, *P *< .001) (Figure 2F). In particular, KLK6 protein expression was not significantly higher in extra-cerebral metastatic samples from patients who had developed brain metastases compared to those who had not.

Overall, the results suggest that *KLK6* overexpression is linked to metastatic localizations in the brain, and to cancer cell implantation in the brain parenchyma whatever the cancer type. However, because the metastatic groups were unmatched and their composition differed across sites and centers, causal attribution of the observed transcriptional differences specifically to brain tropism should be interpreted with caution. We therefore performed additional experimental procedures.

### Modelling the Brain Metastatic Spread in Mice: Is KLK6 an Adaptive Factor?

We modeled the brain metastatic spread in mice with an intracardiac injection of cancer cell lines, as previously reported.^21^^,^^23^^,^^25^ For 3 cell lines, we purposely chose murine cell lines injected in NMRI nude mice considering that murine KLK6 overexpression is required for an adaptive implantation of metastatic cancer cells in the brain.

After an intracardiac injection of cancer cell lines (*N* = 5 animals for each cell line), the mice were euthanized after a median time of 12 days for 4T1 cells, 19 days for B16-F10 cells, 45 days for S49.1 cells, and 42 days for MDA-MB-231 cells. At the time of euthanasia, all the (organs were collected to assess metastatic spread. All the mice developed brain metastases, distributed across both the brain parenchyma and the meningeal area. For the 4 cell lines, extra-cerebral metastases were distributed across various organs including the lung, the liver, the spleen, the ovaries and the kidneys. For 4T1, B16-F10, and MDA-MB-21 cell lines, lung metastatic localizations were the most common, while they were mainly distributed in the spleen for S49.1 cell line.

Basal mRNA expression level of *KLK6* was considerably different for each cell line used. In particular, *KLK6* was overexpressed in S49.1 compared to MDA-MB 231 where the expression is almost absent (Supplementary Table S4). For this reason, we did not perform any modulation of *KLK6* expression in our cell lines.

**Figure 3.**
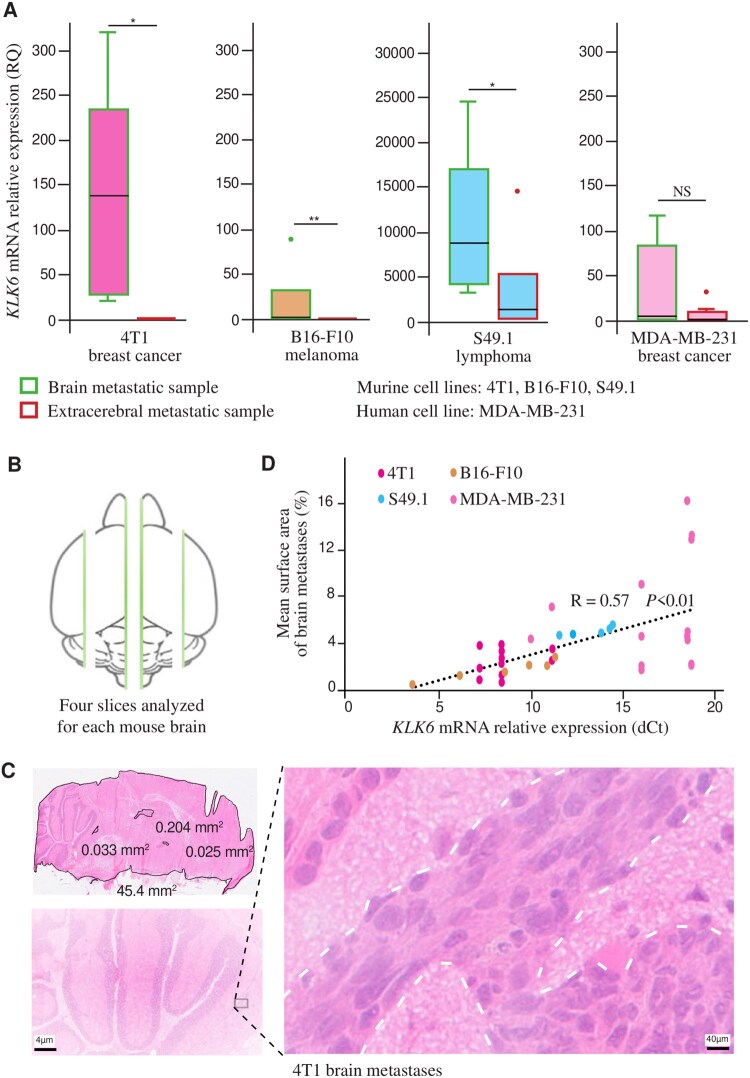
Modeling KLK6 overexpression using murine models of brain metastases. (A) For 4T1, B16-F10, and S49.1 cell lines, KLK6 mRNA expression is significantly higher in brain metastases compared to extracerebral metastases (RQ = 140.1 vs 0.5, *P* = .014 for 4T1, 2 vs 0, *P* = .006 for B16-F10 and 8560 vs 1269.6, *P* = .03 for S49.1). For MDA-MB-231 cell line, the difference is not significant; *P < .05, **P < .01. (B) Schematic representation of the 4 slices analyzed for each mouse brain. (C) This panel illustrates how to quantify the mean surface area of brain metastases on a longitudinal brain section 12 days after intracardiac injection of 4T1 cells in a nude mouse. It is calculated as a ratio of total metastatic area (delineated by white dotted lines) to total brain surface area. (D) Correlation between KLK6 mRNA expression and the mean surface area of brain metastases. The Pearson correlation coefficient is calculated between KLK6 mRNA expression and the mean surface area of brain metastases for corresponding mouse models (*y* = 0.45x − 1.3827; *R* = 0.57; *P* < .01). Each color represents one cell line.

For each mouse, we assessed *KLK6* mRNA expression in laser-microdissected cancer cells from brain and extra-cerebral metastatic samples. For the 3 murine cell lines (4T1, B16-F10, and S49.1) and considering each cell line as a reference, *KLK6* mRNA expression was significantly higher in brain metastases than in extra-cerebral metastases, thus in line with what we had observed in patients. For 4T1, RQ = 140.1 vs 0.5, *P *= .014; for B16-F10, RQ= 2 vs 0, *P *= .006; and for S49.1, RQ = 8560 vs 1269.6, *P *= .03 (Figure 3A). In contrast, for the human cancer cell line MDA-MB-231, *KLK6* mRNA expression was not significantly different in brain metastases from expression in extra-cerebral metastases (RQ = 5.2 vs 3.6, *P *= .6) (Figure 3A).

We then assessed the correlation between *KLK6* mRNA expression and the mean surface area of the brain metastases. For each mouse, the brain was cut into 4 slices (Figure 3B), and metastatic extent was calculated as a ratio of the total metastatic area to the total brain surface (Figure 3C). In parallel, *KLK6* mRNA expression was assessed in laser-microdissected cancer cells for each slice where metastases were identified. For all 20 mice injected with any cancer cell line corresponding to a total of 80 brain sections, there was a significant correlation between *KLK6* mRNA expression and the mean surface area of brain metastases (*R* = 0.57, *P *< .01) (Figure 3D).

### Coculture of 4T1 Murine Cancer Cells With Murine Oligodendrocytes Increased KLK6 mRNA Expression in 4T1 Cells

**Figure 4.**
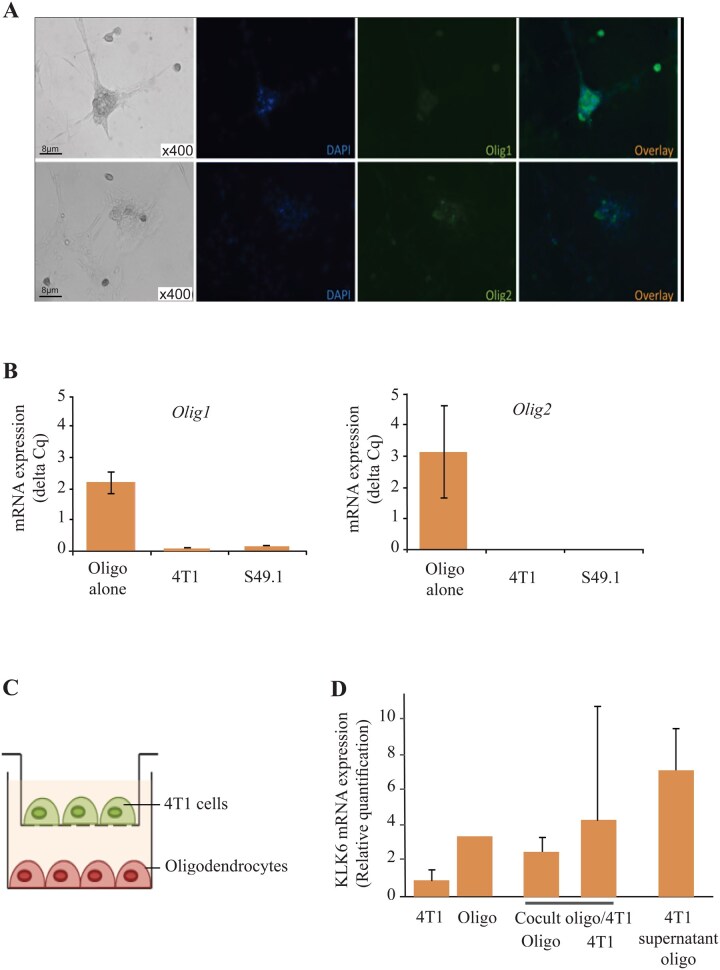
Oligodendrocyte characterization and 4T1 cocultured with murine oligodendrocytes or conditioned medium. (A) Oligodendrocyte immunofluorescence staining using anti-Olig1 (upper panel) and anti-Olig2 (lower panel) antibodies. ×400 magnification. (B) Olig1 and Olig2 are overexpressed in oligodendrocytes compared to 4T1 and S49.1 cells, using RT-qPCR. (C) Schematic representation of Boyden chamber for coculture assay. (D) KLK6 mRNA expression in 4T1 or oligodendrocytes according to different conditions.

Oligodendrocytes were obtained from brains of NMRI mice and cultured in dedicated medium. After 4 days of culture, using immunofluorescence and RT-qPCR, we confirmed that these cells expressed Olig1 and Olig2 oligodendrocyte markers (Figure 4A and B). As oligodendrocytes physiologically express KLK6, we hypothesized that the interactions between cancer cells implanted into the brain and oligodendrocytes could explain the adaptative process of *KLK6* increased expression by metastatic cancer cells. We thus cocultured for 3 days our murine oligodendrocytes with murine 4T1 breast cancer cells using Boyden chambers (Figure 4C). We then evidenced a 2-times increased of *KLK6* mRNA expression compared to native 4T1 cancer cells. In contrast, *KLK6* mRNA expression in oligodendrocytes was not modified. In addition, when 4T1 cells were cultured in modified medium with oligodendrocyte culture supernatant, *KLK6* mRNA expression also increased and was 3-times greater than baseline level (Figure 4D).

Overall, these results suggest that KLK6 overexpression in brain metastases could be an adaptive factor linked to cancer cell implantation in the brain.

## Discussion

In this study on patients and murine models, we demonstrated that KLK6 overexpression was linked to brain metastatic implantation in breast cancers, melanomas, and lymphomas.

KLK6, also known as hK6, neurosin, protease M, or zyme, is encoded by the *KLK6* gene and is a serine protease with trypsin-like properties. It is physiologically expressed in a wide range of tissues, with the highest expression in the central nervous system. In particular, KLK6 is strongly expressed in the luminal cells lining the choroid plexus, in the oligodendrocytes and the brain endothelial cells of gray matter, and in the peripheral nerves.^35^^,^^36^ In the 29 human brain metastases in our clinical study, the *KLK6* mRNA overexpression, as well as the brain signatures that we found, were not linked to this physiological KLK6 expression or to a contamination with normal brain tissue. Indeed, all samples were laser-microdissected to exclusively select cancer cells and prevent any contamination between the tumor microenvironment and normal tissue, which is a strength of our study. We have previously demonstrated the feasibility of performing relevant whole transcriptomic studies on laser-microdissected frozen metastatic samples.^13^^,^^14^

We confirmed that *KLK6* mRNA overexpression in brain metastases was associated with KLK6 increased protein overexpression by cancer cells. This shared overexpression of KLK6 in brain metastases from breast cancer, melanoma, and non-Hodgkin lymphoma has never been reported. Our approach is original, since most studies usually focus on a single cancer type, and data on shared signatures between cancer types is thus limited. In previous studies, some pathways are identified as key regulators in brain metastasis by performing transcriptomic analysis of brain metastases across histology.^37–40^ According to the seed-and-soil hypothesis, the successful growth of metastasis depends on the interactions between cancer cells (seeds) and the metastasized organ (soil). For an implantation to take place, circulating cancer cells need a “suitable soil,” and less than 0.01% of them succeed in forming metastases.^41^ Different cancer types and sub-types have distinct preferential metastatic sites. The gene expression profile of metastatic cells is a key determinant for the formation of site-specific metastases.^42^^,^^43^ But determining whether there are specific signatures linked to organ-ecific metastatic sites remains unclear.^44^ Using public transcriptomic databases, Zhang et al identified a brain metastasis signature common to lung and breast cancers, while normalizing their data using a “normal brain signature.”^45^ In our study, we evidenced that the 23-gene signature, including KLK6 overexpression in brain metastases seems related to an adaptive process of metastatic cancer cells, mimicking the “normal biology” of the metastasized organ, and facilitating their implantation. In our pre-clinical model, we also confirmed that KLK6 overexpression was linked to metastatic implantation in the brain using murine versus human cell line models. Since KLK6 protein homology is only 68% between humans and mice, our results are in favor of an intraspecies adaptive process of KLK6 overexpression after brain implantation of metastatic cancer cells. Using coculture of murine breast cancer cells with murine oligodendrocytes that physiologically express KLK6, we showed that this adaptative process may be linked to a paracrine interaction between tumor cells and oligodendrocytes.

Another strength of our study is that we performed transcriptomic analyzes on brain metastatic samples, and compared them to transcriptomic data from extra-cerebral metastases. We identified 23 genes associated with a brain metastasis signature. In a meta-analysis of genomic alterations in breast cancer brain metastases, we have already underlined the added value of obtaining biopsies from brain metastases to fully explore their biology.^12^ In this meta-analysis, we identified a panel of 6 genes with higher mutation prevalence in brain metastases than in extra-cerebral metastases, which is of particular interest for their potential role in the brain metastasis process. Brain metastases can derive from a minority clone within a primary tumor or from another metastatic localization,^46^^,^^47^ and there have been few transcriptomic studies obtained from brain metastatic samples, most of them comparing transcriptomic data from primary tumors and brain metastases (Supplementary Table S5).

Interestingly, in our preclinical model, we found that a higher *KLK6* mRNA expression in brain metastases was also associated with a larger brain metastatic surface area, suggesting that KLK6 overexpression not only contributes to the adaptive brain implantation of cancer cells, but also to the progression of the disease itself. In vitro studies have shown that KLK6 breaks down fibrinogen and collagens type I and IV, which are components of the basement membrane,^33^^,^^48^ contributing to the invasion and migration of cancer cells.^32^^,^^34^^,^^49–52^ Furthermore, KLK6 overexpression has been reported to increase cancer cell proliferation,^32^^,^^34^ and cell resistance. Among patients with glioblastoma, KLK6 overexpression is associated with an unfavorable prognosis, linked to apoptosis resistance.^28^

In this study, we identified the fact that KLK6 overexpression was linked to brain localizations, which could be a promising strategy for the use of anti-KLK6 for these challenging localizations, regardless of their organ of origin, pending specific cancer cell targeting to avoid cytotoxicity on normal brain cells.

## Supplementary Material

vdag125_Supplementary_Data

## Data Availability

The data that support the findings of this study is available at the following Zenodo repository: [https://doi.org/10.5281/zenodo.18678201].
