# Supplementary material for: A common signature of brain metastases among patients with breast cancer, melanoma, and lymphoma
Source: Neurooncol Adv. 2026 May 10;8(1):vdag125. doi: 10.1093/noajnl/vdag125 (PMC13221969; doi:10.1093/noajnl/vdag125)
Supplement: vdag125_Supplementary_Data [file vdag125_supplementary_data.zip › Supplementary Figure legends.docx]

**Supplementary Figure 1**. Mean RNA Integrity Numbers (RIN) of cancer samples. The mean did not vary according to cancer type.

**Supplementary Figure 2**. Unsupervised Principal Component Analysis (PCA) of transcriptomic data from 133 metastatic samples.

**Supplementary Figure 3.** Analytical workflow for transcriptomic data from metastases.

A. A Kruskal–Wallis test was applied to brain metastases from breast cancer, melanoma, and lymphoma to identify common genes not significantly differentially expressed among the three cancer types.

B. Comparisons between brain metastatic samples and extra-cerebral metastatic samples for each cancer type to identify common genes up-regulated in brain metastases of breast cancers, melanomas, and lymphomas

**Supplementary Figure 4.**

A. Volcano plot from the multivariable binomial GLM of gene expression by metastasis location (brain vs. extra-cerebral), adjusted for tumor type and center. All genes in the previously defined 23-gene signature fall within the red region; KLK6 is labeled.

B. MA plot from the multivariable binomial GLM described in the Methods (brain vs. extracerebral), adjusted for tumor type and center. All genes in the predefined 23-gene signature fall within the red region; KLK6 is labeled.

**Supplementary Figure 5**. Supervised Principal Component Analysis using the 23 genes identified as upregulated in brain metastases. The first three components summarize the variability of the 23 genes and separate brain metastases from extra-cerebral metastases.

**Supplementary Figure 6.** AQP4 mRNA expression according to subgroups in the 3 cancer types.

AQP4 mRNA expression among the three groups (A), and then separately in each cancer type (B, C, D); ** p < 0.01, *** p < 0.001, **** p < 0.0001.
